# The prevalence of risky sexual behaviors among youth center reproductive health clinics users and non-users in Addis Ababa, Ethiopia: A comparative cross-sectional study

**DOI:** 10.1371/journal.pone.0198657

**Published:** 2018-06-07

**Authors:** Nigusie Fetene, Wubegzier Mekonnen

**Affiliations:** School of Public Health, College of Health Sciences, Addis Ababa University, Addis Ababa, Ethiopia; International AIDS Vaccine Initiative, UNITED STATES

## Abstract

**Background:**

Risky sexual behaviors adversely affect the health of youth and young adults exposing them to sexually transmitted infections including HIV/AIDS and unwanted pregnancy to females that in turn lead to deleterious health, social and economic consequences. Youth centers inform their clients on sexually transmitted diseases, including HIV/AIDS, unwanted pregnancy, high risk abortion, and other reproductive health problems. Therefore, this study was designed to assess the prevalence of risky sexual behaviors among youth center reproductive health clinic users and non-users in Addis Ababa.

**Methods:**

A comparative cross-sectional study design was carried out among 524 youth in Addis Ababa from March to April, 2016. The data was entered in EPI-INFO 7 software; and cleaned and analyzed using SPSS version 16.0. The prevalence was computed. Binary and multivariable logistic regression was done to determine the strength, direction and significance of association between youth center reproductive health clinic utilization and risky sexual behavior and to control confounder variables respectively.

**Results:**

A total of 524 youth with the response rate of 92% participated in the study. The overall prevalence of risky sexual behavior was 226 (43.1%) (With statistically significant difference in prevalence among users 101 (38.5%) and non-users 125 (47.7%) of youth center clinics, (p-value = 0.04). The odds of reporting risky sexual behavior was 60% higher among volunteers who did not use the reproductive health clinic, relative to those who did (AOR = 1.60; 95%CI = 1.08, 2.37). Teenagers aged 15–19 years were (AOR = 0.08; 95%CI = 0.05, 0.15) 92% less likely to practice risky sexual behavior compared to those aged 25–29 years old.

**Conclusion:**

Risky sexual behavior was statistically significantly higher among non-users of the youth center reproductive health clinic compared with the users. In addition, a substantial proportion of the youth engaged in different risky sexual behaviors that are evidenced by the existence of multiple sexual partners, sexual practice without condom and early sexual debut that might predispose youth to STIs including HIV infection and unwanted pregnancy. The ministry of health and its partners should strengthen youth center reproductive health clinics in urban, semi-urban and rural parts of Ethiopia.

## Introduction

Although there is no international consensus, the United Nations (UN) defines the youth as individuals between 15 and 24 years of age [1]. The UN estimated that around 1.2 billion people in the world are the youth aged 15–24 years which accounts 17.6% of the total population. More than 80% of them live in developing countries [2, 3]. Youth make up the greatest proportion of the population in Sub-Saharan Africa (SSA), with almost one-third of the population between the ages of 15 and 24. Sub- Saharan Africa is the only region of the world in which the number of youth continues to grow substantially [4].

Ethiopia is one of the least developing countries with a population dominated by the youth aged 15–24 years. According to the 2007 Ethiopian census, the youth aged 15–24 years were more than 15.2 million which was 20.6% of the total population [5, 6]. The Ethiopian government has a different age bracket for the youth (15–29 years) as defined by the Ministry of Youth and Sports which is employed in this study.

Risky sexual behaviors are defined as individual’s sexual practices that may increase the susceptibility of person to the risk of sexually transmitted infections (STIs) including HIV/AIDS, unplanned pregnancies and psychological disorders. Based on the review of published articles some of these behaviors include unprotected sexual intercourse, having multiple sexual partners, early sexual initiation, sexual intercourse with commercial sex workers and bartering sex for money, goods or other favors. Studies have identified that lack of knowledge about undesired consequences of these risky sexual behaviors coupled with poverty were factors that increased the chances of adolescents engaging in risky sexual practices [3, 7, and 8].

Globally, it is estimated that around one—third (38%) of all pregnancies are unintended and studies have indicated that risk factors for unintended pregnancy in Arab countries include early marriage, low socioeconomic status, low education and certain local socio—cultural factors [9]. Furthermore, unwanted pregnancies (either mistimed when they occur or unwanted at all) in the Middle East and North Africa countries (MENA) were estimated between 15% and 58% [10]. Unwanted pregnancies among the youth and complications of induced abortions are among the important health problems in the world. Approximately 20 million abortions are performed worldwide each year of which 95% is practiced in developing countries [11].

Other studies have also reported risky sexual behaviors as a common practice among youth in Sub-Saharan Africa (SSA). Youth in this region were frequently engaged in pre-marital sexual intercourse, with ill consequences such as unwanted pregnancy [12], Sexually Transmitted Infections (STIs) [13], and HIV/AIDS [14, 15]. Against the well accepted cultural norms in Sub-Saharan Africa, the youth also tend to engage in having multiple sexual partners [13–15], concurrent sexual partners [5] and unprotected sexual intercourse [12–15]. Globally, young people aged 15–24 years accounted for 42% of new HIV infections and nearly 80% of them lives in Sub-Saharan Africa. Unless appropriate age and institution targeted intervention exist, certain behaviors can put the youth at a greater risk of HIV infection. Groups of people who engage in these high-risk sexual behaviors are considered as vulnerable to HIV infection and need to be watched cautiously in order to control the epidemic [16]. A study conducted in Moshi, Tanzania secondary schools indicated that 21.6% of them started sexual intercourse before the age of 15 years and from the study participants who ever had sexual intercourse more than 80% started sexual intercourse below the age of 18 years [17–20]. More than 35% had started sexual intercourse before the age of 18 years [21]. According to 2011 EDHS, 29% of women had first sexual intercourse before age 15 years old and 62% of women before the age of 18 years old [22]. Among the respondents who ever had sexual intercourse 27.4% initiated sex before the age of 18 years [23].

Majority of SSA youth have multiple sexual partners in their lives which put them at risk for STIs and unintended pregnancies [24]. A study conducted in Moshi, Tanzania secondary schools showed that 29% of study participants were sexually active. Of which, 47.7% have had more than one sexual partners [17]. The studies conducted in different parts of Ethiopia showed that among those who have had sexual intercourse more than 45% of them having two and more than two sexual partners in the past 12 months [18, 20, 25 and 26]. The studies conducted among West Gojjam zone in-school youths and Mizan-Tepi University students showed that 25% and 42.1% of the respondents from those who ever had sexual intercourse had multiple sexual partners respectively [19, 27]. A study conducted among high school students in Gurage zone of Agena district in Ethiopia showed that 70.5% of the respondents had two or more sexual partners in the past one year of the survey. The mean age of sexual initiation was 16.2 years which is a risk factor for Sexually Transmitted Infections (STIs), unwanted pregnancies and other reproductive health related complications [28].

In Ethiopia limited access to targeted RH care and services for youth contributes to the high burden of RH problems. Over a quarter of all pregnant youth feel that their pregnancies are mistimed, reflecting this population’s limited access to FP and RH services. These unwanted pregnancies entail significant risks for maternal health, including high rates of delivery-related complications and high abortion rates. Unwanted pregnancy is one of the major RH challenges faced by the youth in Ethiopia. About 54% of pregnancies to girls under the age of 15 years were unwanted compared to 37% for those aged 20–24 years [2].

However, no study has been conducted to measure the prevalence of risky sexual behavior among users and non-users of youth center RH clinics in Ethiopia. This study is therefore tasked to measure the prevalence of risky sexual behaviors among the two youth groups in Addis Ababa, Ethiopia.

## Methods

### Study area and period

The study was conducted in Addis Ababa, the capital city of Ethiopia. Addis Ababa is administratively divided into ten sub-cities and 116 districts. The Youth Center is a social and recreational center intended primarily for use by the youth. The Center supports opportunities for youth to develop their physical, social, emotional, and cognitive abilities and to experience achievement, leadership, enjoyment, friendship, and recognition. It also offers organized instructional programs like SRH services. There are a total of 106 youth centers, of which 84 are functional that render services including library, internet, cafeteria, Digital Satellite Television, sport games, different type of trainings, hall renting service, shower rooms to be used by youth, reproductive health services such as Voluntary Counseling and Testing (VCT) of HIV/AIDS, condom distribution, family planning service provision, information on reproductive health, peer education, reproductive health provision by a health care provider. These services have been given since 2011. This particular study was conducted during March to April, 2016.

### Study design and participants

A comparative cross-sectional study design was used. In this study, the comparative groups were the youth who uses reproductive health clinics in the youth center and those who didn’t use such facilities. The intervention group was youth center reproductive health clinics users enrolled and utilized at least one of the RH services and registered in the log books that were randomly selected. The youth that were recruited as an intervention group helped to identify comparative groups in their neighborhood with the same sex and age bracket. The study population was usual resident (at least 6 months) youth in the age range of 15 and 29 years in Addis Ababa. Youth who were severely sick and suspected of mental health problem and those who were unable to hear or speak, involuntary to participate and those who did not have friend in either of the study arm were excluded from the study.

### Sample size determination

The sample size was determined by using double population proportion formula. Taking current prevalence of risky sexual behaviors 45.6% from study done among non-users of the youth center RH clinic [26] to obtain maximum sample size at 95% certainty and a maximum discrepancy of ± 5% between the sample and the population were considered [26]. Having sex with multiple sexual partners was also assumed to be 15% lower among youth center RH clinic users. A power of 80%, 15% non- response rate and 1.5 design effect was also considered. The proportion of users to non-users of youth center reproductive health clinics is one. Then, the final sample size for this study was estimated to be 570.

### Sampling

Multi-stage sampling procedure was used to select study participants. Five of the ten sub-cities in Addis Ababa were randomly selected. Two districts from each of the selected sub-cities that have organized registration book for reproductive health clinic users were selected. Each district has only one youth center reproductive health clinic for which the sample size was proportionally allocated. Therefore, ten study sites were selected by using simple random sampling method. Finally, 524 youth were participated in this study.

### Data collection methods and instruments

The data were collected by using interviewer-administered questionnaire. Structured questionnaire was developed by reviewing different literatures on risky sexual behaviors [28, 29]. The questionnaire was first prepared in English language and then translated to Amharic and back translated to English to check its consistency. Ten clinical nurses working in the youth center RH clinic were trained intensively for two days to collect the data.

### Study variables

#### The main outcome variable

Risky sexual behavior. In this study it is defined as one of the following: never using condoms, inconsistent use of condom, incorrect use of condom, having more than one sexual partner, starting sex before 18 years, being commercial sex worker or having sex with commercial sex workers. Youth center RH clinic services utilization status could be considered as an independent variable. Other socio-demographic variables including; age, sex, income, marital status and educational status, were also observed.

### Data quality assurance

The collected data were checked for its completeness and consistency on daily basis. The quality of data was assured through training of data collectors and close supervision at the time of data collection. Pilot testing was also done in 5% of the sample size in a similar context and population which is not included in the actual study. Five percent of the data was double-entered in order to compare and assure the quality of the data.

### Data analysis and interpretation

Data were entered into Epi-Info version 7 and later exported to and analyzed by using SPSS version 16.0 (SPSS Inc., Chicago, IL, USA). A composite variable for measuring risky sexual behavior was computed by combining early age at sexual debut (under the age of 18 years), having more than one sexual partner, inconsistent or incorrect use of condom, sexual act with commercial sex workers (for males only) and being commercial sex worker (for females only) in the past 12 months from the study period. Frequencies for all variables were counted and cross tabulated using percentages. Binary logistic regression model was used to determine the association between risky sexual behavior and different independent variables, the main one being utilization of youth center reproductive health clinics. Furthermore, multivariable logistic regression analysis was used to see the net effects of each independent variable in explaining variation in the outcome variable. Odds ratio along with the 95% confidence interval was used to ascertain association, statistical significance and direction of association between variables. Statistical significance was set at a p-value of <0.05.

### Ethical consideration

Ethical clearance was obtained from the Research Ethics Committee of the School of Public Health in College of Health Sciences of Addis Ababa University. Official permission was secured from Addis Ababa City Administration health bureau, Sub-cities’ health office’s and districts’ health offices. Verbal consent from each participant was obtained after explaining the purpose of the study. For the participants who were under 18 years, consent was obtained from the parents or guardians. The right of participants to refuse or not to respond to questions they don’t feel comfortable with or discontinue participation at any time was ensured. Confidentiality was kept at each step of the data collection and then after. To assure confidentiality no name or personal identifying information was written on the questionnaire and information was recorded anonymously.

## Results

### Socio-demographic characteristics of study participants

The distribution of study participants by their various socio-demographic characteristics and reproductive health clinic utilization status are shown in Table 1. Of the 570 youth identified as eligible to participate, 524 (92%) gave consent and participated. One-third 185 (35%) of the study participants were females. Two hundred nineteen study participants (42%) were aged between 20 and 24 years with a mean age of 21 (±3.49) years. With regards to ethnicity, 195 (37%) of the respondents were Amhara and 481 (92%) of them were not married at the time of study. Regarding educational status, a total of 434 (83%) respondents were attained secondary school education and/or higher level. More than half, 302 (58%) of the respondents family average monthly income were less than 3001 ETB. Moreover, the distribution of study participants by different socio-demographic characteristics did not show significant difference between youth center reproductive health clinic users and non-user.

**Table 1:** Socio-demographic characteristics of youth by their RH clinic utilization status in Addis Ababa, Ethiopia, 2016.

Socio-demographic characteristics	Category	Number (Percentage)		P-value
		RH clinic users (n = 262)	RH clinic Non-users (n = 262)	
Sex	Female	89 (34%)	96 (37%)	0.583
	Male	173 (66%)	166 (63%)	
Age	15–19	110 (42%)	99 (38%)	0.162
	20–24	99 (38%)	120 (46%)	
	25–29	53 (20%)	43 (16%)	
Ethnicity	Amhara	106 (40%)	89 (34%)	0.683
	Guragie	24 (9%)	30 (11%)	
	Oromo	62 (24%)	66 (25%)	
	Tigrie	32 (12%)	37 (14%)	
	Wolaiyta	17 (7%)	15 (6%)	
	Others*	21 (8%)	25 (10%)	
Marital status	Currently married	22 (8%)	21 (8%)	1.000
	Not in union	240 (92%)	241 (92%)	
Educational status	No education	14 (5%)	20 (7%)	0.458
	Primary education	25 (10%)	31 (12%)	
	Secondary education or higher	223 (85%)	211 (81%)	
Family average monthly income (ETB)	≤1000	29 (11%)	24 (9%)	0.390
	1001–3000	124 (47%)	125 (48%)	
	3001–5000	81 (31%)	73 (28%)	
	≥5001	28 (11%)	40 (15%)	

*Afar, Gamo, Sidamo, Siltie; ETB = Ethiopian Birr.

### Risky sexual behaviors of youth in Addis Ababa

Disaggregation of study participants by their reproductive health characteristics revealed that 254 (48%) of study participants had started sexual intercourse. Among those non-users of the youth center RH clinic, 139 (53%) of them were sexually active compared to those youths who are users 115 (44%) of the RH services in the youth center and the difference was statistically significant. This finding tell us those youths who did not enrolled and utilized the RH services at the youth center were already sexually active. But users of the youth center RH clinic were not sexually active as compared to non-users of the youth center RH clinic. The overall prevalence of risky sexual behavior was 226 (43%) while it was 101 (38%) among youth center reproductive health clinic users and 125 (48%) among non-users of such facilities (P = 0.042).

The mean age of sexual debut was 18.56 (±3.02) years and 94 (37%) of whom started it before the age of 18 years. Early initiation of sexual intercourse (before age 18) was 34 (30%) among users of reproductive health clinics while it was as high as 60 (43%) in non-users of these facilities (P = 0.035). On the other hand, 107 (42%) of the respondents had more than one sexual partners in the past 12 months. Disaggregation of the youth by having multiple sexual partners in the past 12 months revealed that 44 (38%) reproductive health clinic users and 63 (45%) non-users had more than one sexual partners though the difference was not statistically significant.

Besides, 119 (47%) of the respondents did not use condom during their sexual intercourse in the past 12 months. The practice of condom use among youth center reproductive health clinic users and non-users was not statistically significantly different. Condoms have never been used by 48 (42%) of the youth center clinic users while 71 (51%) did not use condom while having sex among non-users of youth center reproductive health clinics in the past 12 months prior to the survey (**see** Table 2).

**Table 2:** Risky sexual behaviors of youth in Addis Ababa by youth center reproductive health clinics utilization status, 2016.

Variables		RH clinic utilization status		P-value
		Number (Percentage)		
		RH clinic users	RH clinic Non-users	
Ever had sex (n = 524)	Yes	115 (44%)	139 (53)	**0.044**
	No	147 (56%)	123 (47%)	
Age at first sexual intercourse (n = 254)	<18	34 (30%)	60 (43%)	**0.035**
	≥18	81 (70%)	79 (57%)	
	Mean±SD	18.70±2.947	18.45±3.084	
Number of sexual partners in the past 12 months (n = 254)	None	7 (6%)	12 (9%)	0.295
	One	64 (56%)	64 (46%)	
	Two and more	44 (38%)	63 (45%)	
Used condom in the past 12 months (n = 254)	Yes	67 (58%)	68 (49%)	0.174
	No	48 (42%)	71 (51%)	
Frequency of condom use in the past 12 months (n = 135)	Sometimes	11 (16%)	12 (18%)	0.496
	Most of the time	14 (21%)	9 (13%)	
	Always	42 (63%)	47 (69%)	
Correct use of condom in the past 12 months (n = 135)	Yes	52 (78%)	53 (78%)	1.000
	No	15 (22%)	15 (22%)	
Had sex with CSW in the past 12 months (n = 163)	Yes	12 (15%)	16 (19%)	0.762
	No	65 (85%)	70 (81%)	
Ever been a CSW in the past 12 months (n = 91)	Yes	4 (11%)	3 (6%)	0.645
	No	34 (89%)	50 (94%)	
Had at least one risky sexual behavior* (n = 226)	Yes	101 (38%)	125 (48%)	**0.042**
	No	161 (62%)	137 (52%)	

*Early sexual debut (<18 years), Had more than one sexual partners, never use of condom, inconsistent or incorrect use of condom, had sexual intercourse with commercial sex workers or being a commercial sex worker; Risky sexual behavior (n = 226), because 28 of the respondents were had safe sex among those who ever had sex; Frequency of condom use and correct use of condom (n = 135), because non-users were excluded; Ever had sex with CSW in the past 12 months (n = 163), we considered only male participants; Ever been a CSW and Ever become pregnant (n = 91), only females were concerned for this; CSW = Commercial Sex Worker.

### Factors associated with early sexual debut

Bivariate and multivariable logistic regression analysis were conducted to examine the relationships between early sexual debut and socio-demographic variables, and to control for confounding variables respectively. All variables with P<0.25 in the bivariate analysis were entered to multivariable logistic regression analysis (**as shown in** Table 3). Based on this, youth center RH clinic utilization status, age group, educational status, sex and marital status were statistically significantly associated with early sexual debut among the study participants. After controlling for the effects of potentially confounding variables using multivariable logistic regression; youth center RH clinic utilization status, age group, educational status and sex were statistically significantly associated with early sexual debut (P<0.05). There was no significant statistical association between marital status and early sexual debut (AOR = 2.18; 95%CI = 0.86, 5.55). Non-users of the youth center RH clinic were 2.39 times more likely to have early sexual debut than users of the RH clinic (AOR = 2.39; 95%CI = 1.25, 4.58).

**Table 3:** Bivariate and multivariable logistic regression analysis of early sexual debut among youth in Addis Ababa, Ethiopia, 2016.

Variables		Early sexual debut (n = 254)			
		Yes	No	Crude OR (9% CI)	Adjusted OR (9% CI)
RH clinic utilization status	Non-user	60 (43%)	79 (57%)	1.81 (1.07, 3.05)*	2.39 (1.25, 4.58)**
	User	34 (30%)	81 (70%)	Reference	Reference
Age	15–19	41 (82%)	9 (18%)	10.07 (4.45, 22.79)*	14.96 (5.96, 37.56)**
	20–24	38 (31%)	84 (69%)	20.35 (8.16, 50.71)*	23.07 (8.54, 62.29)**
	25–29	15 (18%)	67 (82%)	Reference	Reference
Educational status	No formal education	13 (48%)	14 (52%)	0.56 (0.25, 1.26)	2.39 (0.59, 9.78)
	Primary education	11 (49%)	12 (51%)	0.57 (0.24, 1.36)	2.56 (1.03, 6.32)**
	Secondary education or higher	70 (34%)	134 (66%)	Reference	Reference
Sex	Female	44 (48%)	47 (52%)	2.12 (1.25, 3.60)*	2.68 (1.41, 5.09)**
	Male	50 (31%)	113 (69%)	Reference	Reference
Marital status	Currently married	8 (19%)	35 (81%)	Reference	Reference
	Not in union	86 (41%)	125 (59%)	3.01 (1.33, 6.81)*	2.18 (0.86, 5.55)

NB: Early sexual debut was adjusted for RH clinic Utilization status, Age, Sex, Educational status and Marital status,

*Significant for Crude OR &

**Significant for Adjusted OR

The youth in the age group of 20 to 24 were 23.07 times more likely to start sexual intercourse before the age of 18 years compared to the age group of 25 to 29 years (AOR = 23.07; 95%CI = 8.54, 62.29) whereas those in the age group 15 to 19 years were 14.96 times more likely to initiate sexual intercourse before the age of 18 years compared to the age group 25 to 29 years (AOR = 14.96; 95%CI = 5.96, 37.56). Regarding to educational status, the youth with primary education level were 2.56 times more likely to have early sexual debut compared to respondents with secondary education or higher (AOR = 2.56; 95%CI = 1.03, 6.32) though it was not statistically significant those with no formal education were (AOR = 2.39; 95%CI = 0.59, 9.78) more likely engage in early sexual debut. Female youth were 2.68 times more likely to have early sexual debut than male (AOR = 2.68; 95%CI = 1.41, 5.09).

### Factors associated with having multiple sexual partners

Bivariate analysis was done to identify factors associated with having multiple sexual partners. To control confounding variables multivariable logistic regression analysis was made by taking the variables with P<0.25 from bivariate analysis. The youth with no formal education were 2.95 times more likely to have had multiple sexual partners compared to the youth with secondary education or higher (AOR = 2.95; 95%CI = 1.21, 7.19). Whereas, the youth with primary education level were not statistically significantly different from youth with secondary education or higher (AOR = 0.79; 95%CI = 0.29, 2.11) with regards to having multiple sexual partners. Concerning marital status, the youth not in marital union were 3.35 times more likely to have had multiple sexual partners compared to married youth (AOR = 3.35; 95%CI = 1.38, 8.12).

Moreover, the youth aged 15–19 years were 71% times less likely to have had multiple sexual partners compared with those youth within the age group of 25–29 years old (AOR = 0.29; 95%CI = 0.12, 0.67). Similarly, the youth aged 20–24 years were 64% times less likely to have had multiple sexual partners compared with those youth who were aged 25–29 years old (AOR = 0.36; 95%CI = 0.19, 0.68). The youth living with only their fathers were 90% times less likely to have had multiple sexual partners compared with the youth living with both biological parents (AOR = 0.10; 95% CI = 0.01, 0.82). Youth living under marital union were 70% times less likely to have had multiple sexual partners compared with those youth living with both biological parents (AOR = 0.30; 95% CI = 0.12, 0.72). The other living arrangements were not statistically significant with having multiple sexual partners as depicted in Table 4.

**Table 4:** Bivariate and multivariable logistic regression analysis for having multiple sexual partners among youth in Addis Ababa, Ethiopia, 2016.

Variables		Multiple sexual partners (n = 254)			
		Yes	No	Crude OR (95% CI)	Adjusted OR (95% CI)
Age	15–19	16 (32%)	34 (68%)	0.39 (0.19, 0.81)*	0.29 (0.12, 0.67)**
	20–24	46 (38%)	76 (62%)	0.50 (0.28, 0.88)*	0.36 (0.19. 0.68)**
	25–29	45 (55%)	37 (45%)	Reference	Reference
Educational status	No formal education	16 (59%)	11 (41%)	2.12 (0.90, 4.80)	2.95 (1.21, 7.19)**
	Primary education	8 (35%)	15 (65%)	0.78 (0.32, 1.92)	0.79 (0.29, 2.11)
	Secondary education or higher	83 (41%)	121 (59%)	Reference	Reference
Marital status	Not in union	93 (44%)	118 (56%)	1.63 (0.82, 3.27)	3.35 (1.38, 8.12)**
	Currently married	14 (33%)	29 (67%)	Reference	Reference
Living with	Father only	1 (9%)	10 (91%)	0.11 (0.00, 0.94)*	0.10 (0.01, 0.82)**
	Mother only	24 (50%)	24 (50%)	1.14 (0.55, 2.36)	1.01 (0.47, 2.14)
	Relatives	8 (31%)	18 (69%)	0.51 (0.20, 1.31)	0.44 (0.16, 1.19)
	Alone	25 (49%)	26 (51%)	1.10 (0.54, 2.24)	0.70 (0.32, 1.53)
	Under marital union	14 (33%)	29 (67%)	0.55 (0.25, 1.21)	0.30 (0.12, 0.72)**
	Both parents	35 (47%)	40 (53%)	Reference	Reference

NB: Had more than one sexual partner was adjusted for Age, Educational status, marital status, and living arrangement,

*Significant for Crude OR &

**Significant for Adjusted OR

### Factors associated with risky sexual behavior

Bivariate analysis was conducted to identify factors associated with risky sexual behaviors. To control confounder variables multivariable logistic regression analysis was done by taking the variables with P<0.25 from bivariate analysis **(**Table 5). Only four variables including youth center RH clinics utilization status, age, educational status and family average monthly income were associated with risky sexual behaviors in the bivariate analysis. During the multivariable logistic regression analysis only three factors; youth center RH clinics utilization status, age and educational status remained statistically significantly associated with risky sexual behaviors.

**Table 5:** Bivariate and multivariable logistic regression analysis of risky sexual behaviors among youth in Addis Ababa, Ethiopia, 2016.

Variables		Risky sexual behavior			
		Yes	No	Crude OR (95% CI)	Adjusted OR (95% CI)
RH clinic utilization status	Non-user	125 (48%)	137 (52%)	1.45 (1.03, 2.06)*	1.60 (1.08, 2.37)**
	User	101 (39%)	161 (61%)	Reference	Reference
Age	15–19	48 (23%)	161 (77%)	0.08 (0.04, 0.14)*	0.08 (0.05, 0.15)**
	20–24	102 (47%)	117 (53%)	0.23 (0.13, 0.40)*	0.21 (0.12, 0.38)**
	25–29	76 (79%)	20 (21%)	Reference	Reference
Educational status	No formal education	26 (76%)	8 (24%)	2.68 (1.10, 6.53)*	2.85 (1.19, 6.81)**
	Primary education	21 (38%)	35 (62%)	0.49 (0.25, 0.97)*	0.98 (0.51, 1.94)
	Secondary education or higher	179 (41%)	255 (59%)	Reference	Reference
Family average monthly income	≤1000	33 (62%)	20 (38%)	2.09 (1.01, 4.35)*	1.82 (0.79, 4.20)
	1001–3000	110 (44%)	139 (56%)	1.00 (0.58, 1.72)	1.06 (0.58, 1.95)
	3001–5000	53 (34%)	101 (66%)	0.67 (0.37, 1.19)	0.79 (0.41, 1.51)
	≥5001	30 (44%)	38 (56%)	Reference	Reference

NB: Risky sexual behavior was adjusted for RH clinic utilization status, age, educational status and family average monthly income,

*Significant for Crude OR &

**Significant for Adjusted OR

There was a negative association between youth center reproductive health clinic utilization status and risky sexual behavior among the youth in Addis Ababa. The odds of reporting risky sexual behavior was 60% higher among volunteers who did not use the health clinic, relative to those who did (AOR = 1.60; 95%CI = 1.08 2.37)

Moreover, teenagers aged 15–19 years were 92% times less likely to practice risky sexual behavior compared with the youth aged 25–29 years old (AOR = 0.08: 95% CI = 0.05, 0.15) and youth aged 20–24 years were 21% times less likely to have risky sexual behaviors than those youth with the age range 25–29 years old (AOR = 0.21; 95%CI = 0.12, 0.38) The youth who had no formal education were 2.85 times more likely to exercise risky sexual behavior than those who attained secondary education or higher (AOR = 2.8: 95% CI = 1.19,6.81) though the statistical significance vanish the youth with primary education (AOR = 0.98; 95%CI = 0.51, 1.94) were less likely to engage in risky sexual behavior.

## Discussion

The prevalence of risky sexual behavior was significantly higher among non-users of the youth center reproductive health clinics compared with users of such health facilities Age and educational status of the youth were possible predictors of risky sexual behavior in this study. More specifically, early sexual debut was statistically associated with reproductive health clinics utilization status, age, sex and educational status of youth. Having multiple sexual partners was associated with age, educational status, marital status and living arrangement of the youth. This study also revealed that a high proportion of study participants were sexually active. It was higher than the studies conducted in Tanzania and Nigeria [17, 30]. In addition to this, our finding was higher than the studies conducted in different parts of Ethiopia [18, 19, 21, 23 and 26]. However, it was lower than the finding of a study among Wollega University students and high school students in East Gojjam zone of Amhara region [20, 25]. This difference might be due to the difference in educational and exposure status of the youth. Regarding to sexuality of the youth, the findings of this study was consistent with the findings of other studies [25, 31].

The prevalence of having multiple sexual partners in this study was lower than the study conducted among secondary school students in Moshi, Tanzania [17]. In this study, the proportion of youth (both users and non-users of the reproductive health clinic) who have had multiple sexual partners was less than the studies conducted in different regions of Ethiopia [18, 20, 25, 26 and 28]. This finding was higher than the study conducted among in-school youth in West Gojjam zone of Ethiopia [19]. The difference might be due to educational as well as cultural differences between the two communities which could be related to the fact that the youth in Addis Ababa is exposed to more Western values. The overall prevalence of having multiple sexual partners in this study was comparable with the study findings conducted among Mizan-Tepi University students [27].

The mean age of sexual debut (18.7 years for users and 18.5 years for non-users) was comparable to the study conducted among Haramaya University students and greater by almost two years for college preparatory students in Guragie zone which was 16.2 years. The difference could be related to the establishment of youth center reproductive health and other interventions in Addis Ababa [21, 28]. The finding from Nigeria secondary school students showed that the mean age of sexual initiation was 14.8 years [30]. The proportion of youth who started sexual intercourse before the age of 18 years in this study was lower that the study finding from Moshi, Tanzania [17]. The possible reason might be differences in socio-cultural and demographic characteristics of the study participants.

The percentage of the youth who started sexual intercourse before the age of 18 years in this study was less (even for youth center reproductive health clinic non-users) than the findings from Benshangul Gumuz region, West Gojjam zone, East Gojjam zone of Ethiopia and Ethiopian Demographic and Health survey (EDHS, 2011) [18–20,32] implying that early age of sexual debut in rural regions of Ethiopia is related to the fact that early marriage is still prevalent in rural regions and sexual debut is predominantly within marriage in these regions. It may also indicate the impact of better RH health messages and information in urban areas like Addis Ababa compared with rural regions. Besides, it could be related to the influence of youth center health services in Addis Ababa and other urban centers though it warrants a detailed qualitative study.

The overall prevalence of early sexual debut in this study was comparable to the finding from Haramaya University students [21] but the prevalence from non-users of the reproductive health clinic was somehow higher than the findings of a study done among Haramaya University students. The difference might be due to educational difference of the two youths. In this study, the magnitude of early sexual debut was slightly higher among both users and non-users of the reproductive health clinic users than the study conducted among Addis Ababa University undergraduate students two years ago [23].

Our study further identified that, female youth were more likely to have early sexual debut than their counterparts. It was comparable with other studies [31, 33]. However, this study finding contradicts with the previous study conducted among high school students in East Gojjam zone documents that males were more likely to have early sexual debut than females [20].

Condom is an important intervention strategy for the prevention of STIs including HIV/AIDS and unwanted pregnancies. Our finding on condom use was consistent with the study conducted in Moshi, Tanzania and World development report [17, 34]. But the prevalence of condom use in this study was higher than the findings in Benshangul Gumuz region and Addis Ababa city [18, 26]. However, this finding was lower than the studies conducted in East Gojjam, and Gurage zones of Ethiopia and South Africa [20, 28 and 29]. The difference might be attributed to differences in the study periods or reproductive health related interventions in the study areas.

In this study, almost half of those who had sexual intercourse in the last 12 months had used condom during their sexual intercourse. This is lower than the findings of the BSS-II and Jimma University students [35, 36]. This might be because of the behavioral interventions that have been given at different levels. But our finding regarding to condom use was almost similar with the finding of EDHS 2011 [22]. The reported low utilization rate of condom use in this study is an indication of the fact that high risk behaviors are still widely practiced among youths in the study area. This calls for a well-organized information, education and communication through peer educators to bring about behavioral change.

One alarming finding in this particular study is that even though no statistically significant difference between users and non-users of the youth center reproductive health clinic, a higher proportion of the sexually active male youth had sexual intercourse with commercial sex workers. This finding is considerably greater than the study done in West Gojjam zone of Ethiopia [19]. In contrast, this finding was lower than the study findings among high school youth in Benshangul Gumuz region and Madawalabu University students [18, 37]. This finding was consistent with the study conducted among Haramaya University students [21].

The overall prevalence of risky sexual behavior in this study was higher than the study conducted among Haramaya University students and high school students in Addis Ababa [21, 26]. However, it was lower than the study conducted among female youth in Tiss-Abay [38]. The difference might be due to methodological differences like; study population, sampling method, sample size and time laps.

This current study illustrates that; youth with age less than 20 years old were less likely to have had multiple sexual partners compared with the youth in the age range of 20 to 24 years old. This finding was comparable with the findings of other studies conducted among Addis Ababa University students [23]. Youth in the age group of 25 to 29 years was more likely to practice risky sexual behavior than those in the age group of both 15 to 19 and 20 to 24 years old. This finding was also consistent with other studies [18, 38]. This study is subject to several limitations. The behavioral outcomes are based on self reported information, which is subject to reporting errors and bias. Since this study touches very sensitive and private issues social desirability bias cannot be ruled out. There is also selection bias in the study sites. Finally, this study was based on cross sectional data, which implies that the direction of casual relationships cannot always be determined.

## Conclusions

This study has shown that a considerable proportion of non-users of the youth center reproductive health clinic youth engage in risky sexual behaviors than users of the youth center reproductive health clinic. The study also shown that majority of youth were engaged in risky sexual behaviors that are evidenced by the existence of multiple sexual partners, sexual practice without using condom, early sexual debut, having sex with commercial sex workers (for males) or being a commercial sex worker (for females) that might predispose to STIs including HIV infection and unwanted pregnancy. In addition to this, reproductive health clinic utilization status, age and educational status of youth are independent predictors of risky sexual behaviors. Therefore, there should be consistent health education message for the wider public to reduce risky sexual behaviors. The urban youth center reproductive health clinics should be strengthened. Moreover, an effort should be made to scale this program in semi-urban and rural communities in Ethiopia.

## Supporting information

S1 Dataset Raw data of some numerical variables in the study*. *Age, income, age at the time of first sexual intercourse, number of sexual partners in the past 12 months, age at the time of first pregnancy.
(XLSX)
